# Reusability of immobilised lipase in the production of omega-3 oils from squid viscera

**DOI:** 10.1007/s00253-026-13799-w

**Published:** 2026-03-27

**Authors:** M. Amdadul Haque, Brendan J. Holland, Colin J. Barrow

**Affiliations:** 1 Centre for Sustainable Bioproducts, School of Life and Environmental Sciences, Deakin University, Waurn Ponds, Australia; 2 Department of Food Engineering, Gazipur Agricultural University, Gazipur, 1706 Bangladesh

**Keywords:** Immobilised lipase, Enzyme reusability, Carrier degradation, Squid viscera oil, Omega-3 oil

## Abstract

**Abstract:**

Squid viscera, a waste product from squid processing, is a marine source of omega-3 fatty acid–rich oil suitable for nutritional supplement use. Squid visceral oil requires extraction and neutralisation of free fatty acids prior to consumption. Immobilised lipase (Lipozyme RMIM) is suitable for green neutralisation with high acylglyceride yields, high oxidative stability and retention of the antioxidant astaxanthin. Long-term utility of this method depends on lipase stability and reusability over multiple reaction cycles. To assess stability, this study monitored the performance of immobilised Lipozyme RMIM over 35 successive cycles of reuse in a custom-built one-litre reactor. This effectively reduces enzyme cost to 2.9% compared to single cycle use costs. We found a maximum of 97% free fatty acids in the crude oil were converted to acylglycerols under optimised reaction conditions in the first cycle, reducing to 86% after 35 reaction cycles. The partial loss of enzyme activity after each cycle appears to be a combination of enzyme unfolding and aggregation with a physical fracturing and breakdown of the resin, particularly after 30 cycles.

**Key points:**

• *Immobilised Lipozyme RMIM neutralises squid oil effectively for 32 repeat cycles*

• *Fatty acid composition of re-esterified squid oil is consistent over repeat cycles*

• *Enzyme denaturation and carrier breakdown led to changes in lipase performance*

## Introduction

Fatty fish and algae are the principal dietary sources of long-chain polyunsaturated fatty acids (PUFAs) including eicosapentaenoic acid (EPA) and docosahexaenoic acid (DHA) (de-la-Haba et al. 2023; Osman et al. 2001; Sahena et al. 2009). EPA and DHA have been shown to be beneficial in the prevention of some diseases, including chronic diseases such as cancer;, high blood pressure, depression, Alzheimer’s, and cardiovascular diseases (Ciriminna et al. 2017; Taati et al. 2018). EPA and DHA are also reported to be effective in lowering plasma triacylglycerides (TAG) and cholesterol, and functional in reducing diabetes mellitus and ischemic heart disease (Bang et al. 1971; Perez-Velazquez et al. 2024). Fish oils are widely used to supplement dietary intake of EPA and DHA; however, increasingly scarce supplies are driving interest in sustainable alternative sources such as microalgae and fisheries by-products (Aitta et al. 2021; Fauziah et al. 2024; Zhang et al. 2021). For example, viscera by-products from squid processing contain approximately 10% oil, which is made up of 13 to 20% EPA and 20 to 30% DHA (Kang et al. 2005; Quispe-Fuentes et al. 2025), making this an excellent source of omega-3 and DHA in particular.

Squid viscera oil can contain up to 45% free fatty acids (FFA) which adversely impact stability and require neutralising to produce a nutritional oil (Kolanowski and Laufenberg 2006; Meivelu Moovendhan et al. 2023; Rodríguez et al. 2021). Conventional neutralisation via alkali refining has drawbacks including low yield due to the removal of the FFA and loss of the antioxidant astaxanthin, naturally present in squid (Mariem and Fatima 2017). Enzymatic glycerolysis of FFA, applied in this study, is a greener alternative method, which results in the incorporation of the FFA into TAG rather than its removal, and has been successfully applied to vegetable, tuna, anchovy and squid oils without loss of yield (Akanbi and Barrow 2015; Baeza-Jiménez et al. 2013; Mustafa et al. 2023; Rubio-Rodríguez et al. 2010). The enzymatic process is favoured due to high catalytic efficiency and mild reaction conditions. In a previous work, we have developed a laboratory and pilot scale method for reduction of squid oil free fatty acid levels from 44 to 4% using immobilised *Rhizomucor miehei* lipase (Lipozyme RMIM) (Haque et al. 2024; Joshi et al. 2025). This method resulted in significantly improved oxidative stability (0.06 to 18.9 h by rancimat) and no loss in astaxanthin content (194.1 µg/g) of the oil after reduction of free fatty acid levels (Joshi et al. 2025). The lipase RMIM used is a 1,3 specific lipase immobilised on a poly(methyl methacrylate) (PMMA) resin carrier by adsorption. This catalyst was selected over the nonregiospecific lipase *Candida antactica* lipase B (CALB) in our earlier work due to a more complete reaction at lower temperatures and thus more energy efficient process (Haque et al. 2024). The regiospecificity of RMIM did not adversely affect yield, despite the sn-2 position not being directly converted. Acyl migration does not have a significant impact on the product oil, which has EPA preferentially distributed at sn-2 and DHA retained at the naturally occurring sn-2 position, which is favourable for oil bioavailability and stability (Joshi et al. 2025). Whilst the method is successful for green neutralisation of squid oil with high acylglyceride yield and high retention of astaxanthin, stability and reusability of the lipase over multiple reaction cycles is unknown. This information is important for viability of the process, which depends on reusing the relatively expensive immobilised enzyme for multiple cycles where the cost can be divided by the number of reuses of the enzyme (Mustafa et al. 2023).

Immobilised enzymes are typically more robust and resistant to environmental changes than free enzymes and are well suited to oil–alcohol (especially glycerol) esterification reactions (Homaei et al. 2013; Sheldon et al. 2021). Our aim in this study was to investigate the reusability and performance of immobilised Lipozyme RMIM for conversion of the high FFA squid viscera oil into a product containing greater than 95% acylglycerols through reaction of FFA with glycerol over successive reuse cycles. We also assessed the loss of activity at each cycle and the reason for activity loss. Understanding the reusability of immobilised Lipozyme RMIM is an important factor to determine the viability of commercial scale omega-3 oil production. Identifying the reasons for loss of enzyme activity can also lead to future improvements in the design of enzyme reactors (Joshi et al. 2025). Previous work with Lipozyme RMIM has shown stability varying from just 2 cycles to 4, 5, 9 and over 20 cycles, depending on the specific reactor design and reaction conditions (Arifin et al. 2012; Castiglioni et al. 2020; Kuo et al. 2012; Nadiah et al. 2024). Therefore, this study aimed to determine the enzyme reusability for neutralisation of oils with high free fatty acid content.

Factors likely to affect enzyme reuse include stability of the enzyme support, strength of the enzyme-support bond and retention of enzyme structure. Normal stirring conditions result in rapid breakdown of the enzyme support, and so to minimise mechanical degradation, our reaction takes place in a custom built one-litre reactor, with an enzyme compartment separated from the overhead stirrer as previously described (Haque et al. 2024). In addition to reaction yield, we monitored qualitative changes to the enzyme over multiple cycles by observing changes in both the enzyme structure and resin properties.

## Materials and methods

### Materials

Crude squid visceral oil was provided by Mantzaris Fisheries Pty Ltd, North Geelong, Australia. The acid value of the crude oil was 62 mg KOH/g (Hassan et al. 2020) and peroxide value 2.1 mEq/kg (AOAC, 2000). Glycerol was purchased from EnviroChem International Pty Ltd, Melbourne, Australia, and immobilised enzyme (Lipozyme RMIM, Novozymes A/S, Denmark; Activity 275 Interesterification Unit/g) from Oppenheimer Pty Ltd, Victoria, Australia. Molecular sieves (3 Å beads, 8–12 mesh), HPLC grade hexane, heptane, copper sulphate, sodium potassium tartrate, acetyl chloride, 8-anilino-1-naphthalenesulfonic acid (ANS), 2,6-di-tert-butyl-4-methylphenol (butylated hydroxytoluene; BHT), methyl nonadecanoate and diethyl ether were sourced from Sigma Aldrich, Castle Hill, Australia. Acetone, sodium hydroxide, sodium deoxycholate, sodium chloride and methanol were purchased from Chem Supply, Gillman, Australia, and toluene from Thermofisher Scientific Australia. TLC standards from Nu-Chek Prep were used to identify each lipid class.

### Enzymatic esterification of squid oil

The esterification of crude squid visceral oil was carried out in a 1-L reactor as previously described (Haque et al. 2024). In summary, 2.5% (m/v) enzyme was initially mixed with oil and glycerol in a 3:1 ratio (m/v) with 1% (m/v) molecular sieves. Additional glycerol (10, 7.1 and 7.1% v/v) was added at 2, 4 and 6 h respectively. The reaction was maintained at 50 °C for 8 h and stirred at 400 rpm. At the end of each reaction cycle, the liquid contents were removed, and the reactor loaded with fresh substrate to repeat the esterification. A total of 35 reaction cycles were completed using the same enzyme in each cycle.

### Modelling enzymatic deactivation kinetics

The deactivation kinetics of the Lipozyme RMIM on a cycle by cycle basis was described by a first-order deactivation model (Kabir and Ju 2023; Zhang et al. 2010). This model was fitted with the real-time conversion performance per cycle. The conversion data at the 12, 18, 24, 30 and 35th cycles were used to calculate the deactivation kinetic rate constant.

According to the first-order deactivation model:

1 $$\begin{array}{l}\frac{dA}{dt}=-K_{in}A\\A=Aoe^{-kint}\;\left(by\;integrating\right)\end{array}$$

2 $$In\left(\frac A{Ao}\right)=-K\mathbf{in}t\;\left(In\;\log arithmic\;form\right)$$

where *A* is the enzyme activity at time *t*, *Ao* is the initial enzyme activity; and *K*_in_ is the deactivation rate constant (cycle^−1^).

### Sampling of enzyme and preparation for analysis

Approximately 12% (m/m) of the enzyme was sampled after 12, 18, 24 and 35 cycles. Excess enzyme was loaded before cycle 1 (2.5% m/v enzyme to substrate as previously stated) to ensure sufficient enzyme in the reactor for the purposes of catalysis and sampling. The excess enzyme was assumed to have no effect on yield according to results from our previous optimisation study (Haque et al. 2024). This study established that an amount of 1% (m/v) enzyme to substrate volume was an optimum concentration and that higher levels did not have a significant effect on the conversion performance of FFA to acylglycerols (Haque). Furthermore, sampling enzyme from the reactor was assumed to have no effect on the reaction yield in each cycle. Enzyme sampling will however progressively affect the enzyme distribution and overall fluid movement inside the reactor. This will potentially increase mechanical stress on the remaining enzyme particles in later reaction cycles and lead to more rapid breakage in the solid support. Thus, enzyme reusability could be slightly underestimated in this study. Before collecting sample enzyme, the reactor was emptied and inverted overnight to allow complete draining of oil and glycerol. The enzyme was trapped inside mesh at the top of the inverted reactor, ensuring the reaction media drained away to avoid any adverse impacts on the enzyme activity. The 12% (m/m) sample of enzyme was collected and washed thoroughly twice using n-heptane to remove any residual oil product and dried at room temperature. A portion of sample enzyme was ground using mortar and pestle. Both the whole and ground enzymes were preserved in brown colour glass vials at 0 °C for further use. The remaining enzyme was used for subsequent reaction cycles without washing.

### Determination of lipid classes by TLC–FID (Iatroscan)

Lipid class analysis was performed using the method described by Akanbi et al., 2013).

### Surface hydrophobicity changes by ANS fluorescence spectroscopy

The change of surface hydrophobicity due to unfolding of the enzyme, due to potential denaturation with each run cycle, was measured by 8-anilino-1-naphthalenesulfonic acid (ANS) fluorescence spectroscopy as previously described (Haque et al., 2013; Joshi et al., 2011). The dried enzyme powders (original and used, whole) were diluted to 1 mg/mL concentration in 0.1 M phosphate buffer (pH 7.0). The fluorescence intensity (FI) and the emission wavelength of ANS-conjugates were measured using a Varian Cary Eclipse fluorescence spectrophotometer. The peak value of the emitted spectrum was used to calculate the relative fluorescence intensity (RFI) for each concentration using Eq. (3):

3 $$RFI=\left(F_S-F_O\right)/\left(F_O\right)$$

where F_s_ and F_o _are fluorescence intensity values of ANS in enzyme suspension and ANS in buffer, respectively.

### Morphological structure observation using light microscopy

Physical changes to the enzyme support material over the reaction cycles were observed using a Zeiss HBO 100 Microscope with attached ZEISS Axio Imager. The video and images were processed using in-built AxioVision 4.7.1 software. Both the heptane-washed and unwashed samples were imaged. For unwashed samples, the adhered surface oil was removed as much as possible using low lint wipes (Kimtech Delicate Task Wipers). The original and used enzymes were imaged both as individual particles and as groups of particles with 5 ×/0.13 magnification.

### Monitoring enzyme structure changes using FTIR spectroscopy

FTIR spectra were obtained in triplicate and analysed to quantify changes in enzyme secondary structural configurations over multiple reaction cycles. FTIR spectra were acquired using a Bruker Alpha-p ATR-FTIR instrument. The sample powders were diluted to 15 mg/mL (m/v) using deionised water containing 100 mM sodium chloride (NaCl) (Haque et al. 2014; Weert et al. 2001). A triglycine sulphate (TGS) detector was used to scan samples from 650 to 4000 cm^−1^. Using a 0.2 cm/sec scanning rate and 4 cm^−1^ resolution, 8 scans in total were collected. The baseline subtracted spectra of samples were analysed by using operated by Opus 7.5 software and PeakFitv4.12 software. A local least square (LLS) algorithm was utilised to fit spectra (of amide region-I, 1600–1700 cm^−1^) without smoothing with Gaussian shape. Secondary structure configurations (α-helix, β-sheet, β-turns and random coil) were approximated using Eq. (4). (Haque et al. 2015; Yazdanpanah and Langrish 2013).

4 $$Secondary\;structure\;\left(\%\right)=Aind/Aall\times100$$

where Aind is the sum of area for all secondary structural individual elements inside amide I band and Aall is the sum of area of all secondary structures inside amide I band.

Secondary structural element band locations were identified as suggested by (Kong and Yu 2007; Natalello et al. 2005). The bands from 1620 to 1640 cm^−1^ were assigned to β-sheets. Random coil was assigned to 1641 to 1647 cm^−1^. Bands from 1648 to 1660 cm^−1^ were assigned to α-helix. Similarly, 1663 cm^−1^, 1671 cm^−1^, 1683 cm^−1^ and 1688 cm^−1^ were allocated to β-turns. The bands near 1691 cm^−1^ were estimated as β-sheets (high frequency). The peaks between 1600 ^−^  to 1619 cm^−1^ were ignored given they are generated from aromatic side chains (Byler et al. 1986; Ngarize et al. 2004). Changes in enzyme secondary structure were quantified using PeakFit V412 band fitting software.

### Modified Biuret test for the presence of enzyme in the squid oil product

The Biuret reagent comprised copper sulphate, sodium hydroxide and sodium potassium tartrate, which produces a deep blue colour in aqueous solution in the presence of the enzyme. The modified Biuret method has been successfully applied in protein quantitation in high fatty samples like the kidney, heart and brain (Beyer 1983). In this study, 2 mL of each oil sample was taken in graduated glass tubes and 3 mL solvent mix (acetone/hexane 1:1) added in the tubes. The tubes were then vortexed for 20 s. Two (2) mL of aqueous biuret reagent and 0.1 mL 10% sodium deoxycholate (pH 8.0) were mixed in the tubes. The tubes were mixed well by hand and kept in boiling water for 30 s or up to full colour development. The contents were then cooled to room temperature in ice and images captured for a qualitative assessment of enzyme leaching into the squid oil products.

### Monitoring product fatty acid composition by GC-FAMEs

The fatty acid compositions of oil samples produced after esterification were determined by gas chromatography-fatty acid methyl esters (GC-FAMEs) using flame ionisation detector (FID) as previously described by (Akanbi and Barrow 2016). The GC fatty acid standards were a mixture of 34 saturated, monounsaturated and polyunsaturated fatty acids in carbon chain length from 4 to 24 purchased from Nu-Chek Prep (Elysian, MN, USA). The internal standard, acetyl chloride and BHT were sourced from Sigma Aldrich, Castle Hill, Australia.

### Statistical analysis

Each analytical test was carried out at least in triplicate, and the average values are reported in the ensuing sections. Data analysis was carried out using in-built software of the instrument, and the mean values and standard deviation of the data were calculated using Microsoft Excel Professional Plus 2019. A 95% confidence level (*p* < 0.05) was applied in these analyses.

## Results

### Enzymatic catalysed esterification reaction yields over multiple cycles

**Fig. 1:**
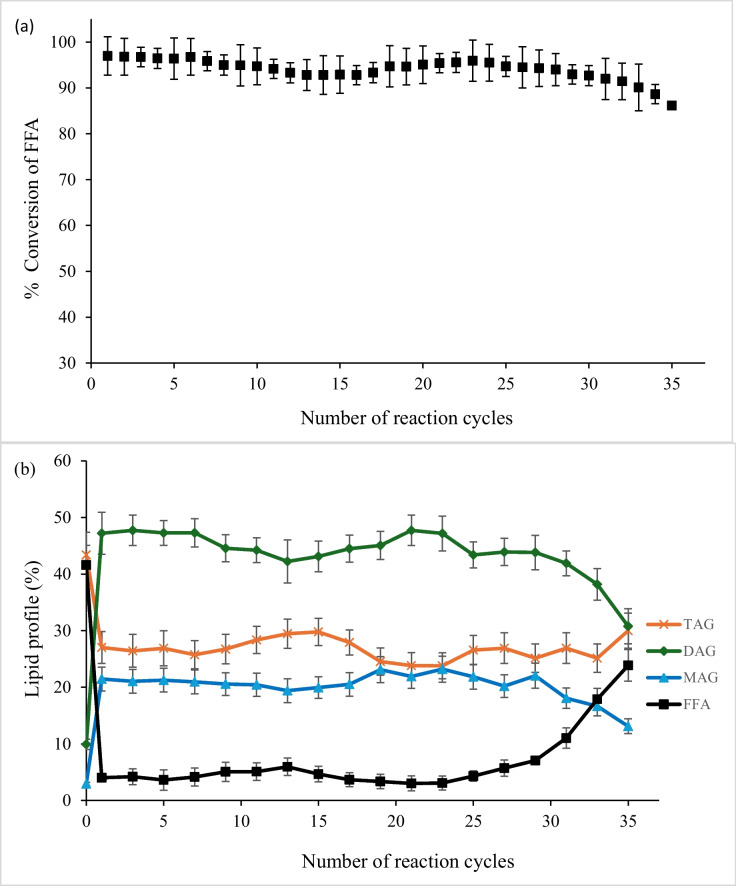
Changes of Lipozyme RMIM performance in neutralising squid viscera oil monitored by (**a**) percentage conversion of free fatty acids over 35 cycles and (**b**) evolution of lipid class profile with each cycle. A significant decline in enzyme activity (*p* < 0.05) was observed from cycle 1 to 35. Error bars represent analytical variability from triplicate measurements of oil profile at each cycle

The supplied crude squid viscera oil contained 41.6% triacylglycerols (TAG), 40.3% FFA, 10.1% diacylglycerols (DAG) and 5.5% monoacylglycerols (MAG). The cycle-to-cycle conversion of FFA to acylglycerols was monitored over 35 reactions, and the results are presented in Fig. 1a, adjusted to represent the FFA content of crude squid oil as 100 percent. After the first cycle, the product oil lipid profile was 27.10% TAG, 3.03% FFA, 48.11% DAG and 21.47% MAG (Fig. 1b) with 96.97% conversion. The reaction yield decreased to 92.81% on cycle 13 (29.46% TAG, 5.95% FFA, 42.23% DAG and 19.4% MAG). Interestingly, the performance of the enzyme then improved after cycle 14, increasing to 95.92% at cycle 23 (23.79% TAG, 3.08% FFA, 47.18% DAG and 23.21% MAG). Performance again gradually decreased to 90.09% at cycle 33 and 86.14% after cycle 35 (30% TAG, 23.86% FFA, 30.78% DAG and 13.12% MAG). FFA content of the product oil was below 6% for the first 27 cycles and then steadily increased to 23.86% after 35 runs. TAG content of the product oil ranged from 23 to 30% after each of the 35 cycles; DAG content had a greater change from 47.2% after the first cycle to only 30.78%, and MAG content dropped from 21.46 to 13.12%. We did not continue analysis after 35 runs due to irreversible degradation of the immobilised enzyme system as described in the next section, significant difference (*p* < 0.05) observed from cycle 1 to 35, and resulting sharp increase in FFA content of the product oil.

### Enzyme deactivation modelling

**Fig. 2:**
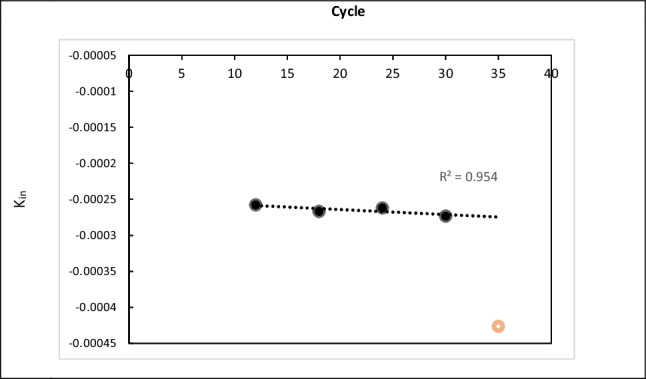
Lipozyme RMIM deactivation rate constant per cycle number

The deactivation rate constant per cycle is presented in Fig. 2. A first-order linear curve is observed over cycles 12, 18, 24 and 30, with a minimal deactivation change rate of K_in_ values ranging from −0.00025 to −0.0003. A more significant fall to −0.00043 is observed at the 35th cycle. The rate constants of each cycle (12, 18, 24 and 30) were linearly fitted according to the trendline with *R*^2^ value of 0.954. The estimated rate constants reflect the conversion performance of our used enzyme up to the 30th cycle and beyond. It is usual that the enzyme deactivation rate constant can change over time due to environmental factors, structural changes in the enzyme or the complexity of the deactivation mechanism (Sadana 1988).

### Changes in enzyme surface hydrophobicity over multiple reaction cycles

**Fig. 3:**
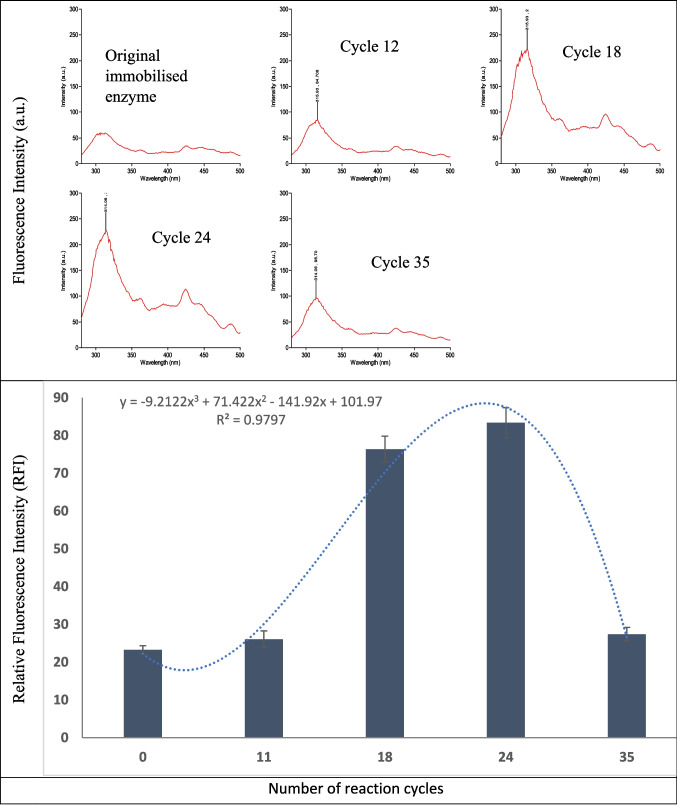
Change of surface hydrophobicity of immobilised RMIM with increasing number of reaction cycles measured using ANS fluorescence spectroscopy. Error bars represent analytical variability from triplicate measurements of immobilised RMIM at each cycle

The ANS fluorescent probe binds to hydrophobic moieties of enzymes in an experimental solution (Deshpande and Sathe 2018; Guliyeva and Gasymov 2020). In the enzyme native state, the hydrophilic portion remains on the surface while the hydrophobic portion is buried, explaining relatively low fluorescent intensity in the native immobilised enzyme (Fig. 3). Relative fluorescence intensity significantly increased over cycles 18 to 24 and then reduced by cycle 35.

The gradual progress to irreversible denaturation of enzyme resulted in the decline in enzyme performance from cycle 24 onwards, presented by a decrease in fluorescence (Fig. 3).

### Morphological changes to immobilised enzyme particles

**Fig. 4:**
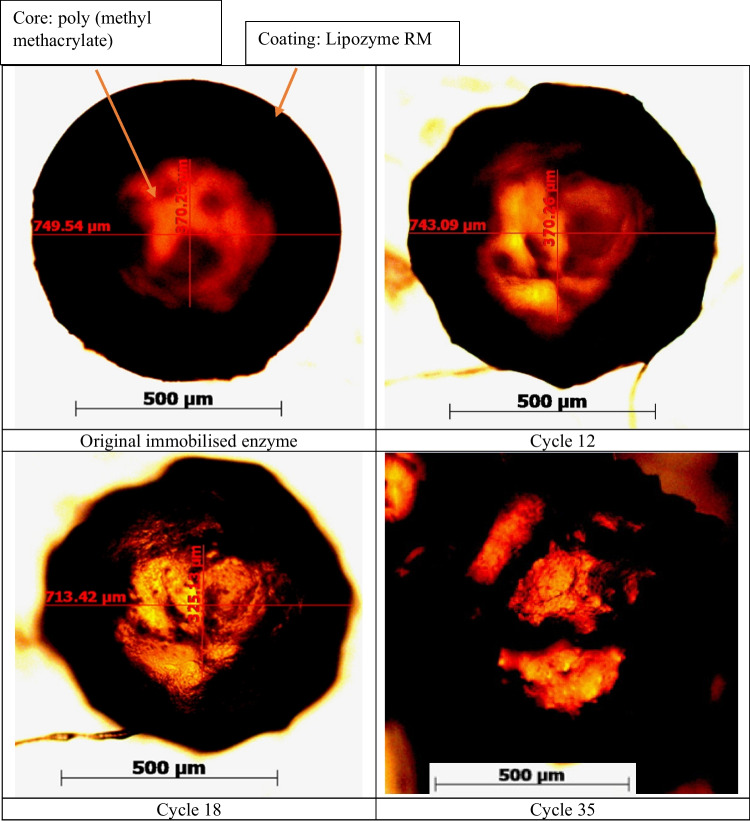
Microscopic images of individual immobilised RMIM enzyme particles with the increasing number of reaction cycles

The structural change of an immobilised enzyme particle with an increasing number of reaction cycles is compared in the microscopic images in Fig. 4. The enzymes collected after 12, 18 and 35 cycles were imaged without washing or drying. Oil adhering to the enzyme was removed, and a minimum of 10 randomly selected particles were examined from different reaction cycles. A representative image from each sampling cycle (cycle 0, 12, 18 and 35) is presented in Fig. 4. The coating of enzyme appears as a black ring surrounding the red-coloured resin core. The diameter of a whole Lipozyme RMIM coated sphere ranges from 500 to 1000 µm and comprises an approximate 55:45 (enzyme/core) thickness ratio. In the cycle 35 image, the enzyme core is fragmented.

**Fig. 5:**
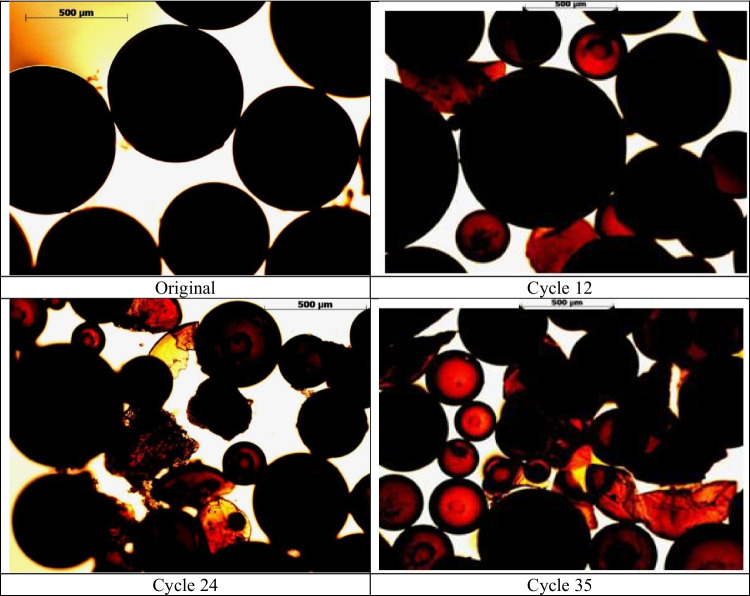
Morphological changes of the enzyme particles at different levels of cycling under a light microscope

Figure 5 shows collections of the immobilised enzyme particles washed with n-heptane and air-dried. The particles were sampled and compared with the original immobilised enzyme. The original enzyme was of smooth spherical shape. However, after 12 cycles, the particles started showing signs of breakage. A significant number of particles were fractured and broken after 24 cycles, and after 35 cycles, irreversible degradation was evident.

### Changes to Lipozyme RMIM secondary structure

**Fig. 6:**
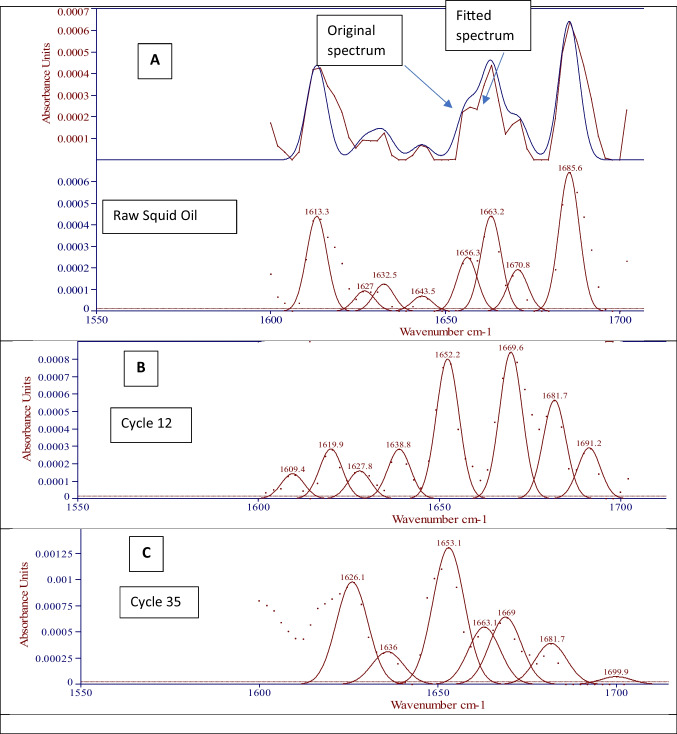
Quantifying the secondary structural elements found on the Amide I region of the (**a**) original (with example of original and fitted spectra) and used enzyme after (**b**) 12 cycles and (**c**) 35 cycles using FTIR spectrum peak fitting software. Each of the above FT-IR spectra was collected in triplicate and is representative of the replicates

Previous work has identified the amide-I region (between 1600 and 1700 cm^−1^) as the most reliable infrared absorbance zone to study and compare secondary structure (Fu et al. 1994; Weert et al. 2001). The apparent changes in secondary structure of these elements of reaction cycles are presented in Fig. 6. The original Lipozyme RMIM secondary structure was calculated to consist of 18.38% α-helix, 15.41% β-sheet, 60.67% β-turns (low frequency) and 5.54% random coil. After 12 cycles α-helix and β-sheet structures increased, compensated by lower β-turns and complete loss of the random coils (27.02% α-helix, 20.61% β-sheet (low-frequency) and 11.26% β-sheet (high-frequency), 41.11% β-turns and 0% random coil). This composition remained relatively constant after 18 cycles, (25.54% α-helix, 21.38% and 10.59% β-sheet, 41.49% β-turns and 0% random coil) and 24 cycles (26.05% α-helix, 20.46% β-sheet, 41.12% β-turns and 0% random coil).

After 34 cycles, the levels of α-helix and β-sheet were even higher at the expense of β-turns (34.21% α-helix, 22.13% β-sheet with a slight decrease of high frequency, 38.34% β-turns and 0% random coil).

### Monitoring enzyme–resin adhesion

**Fig. 7:**
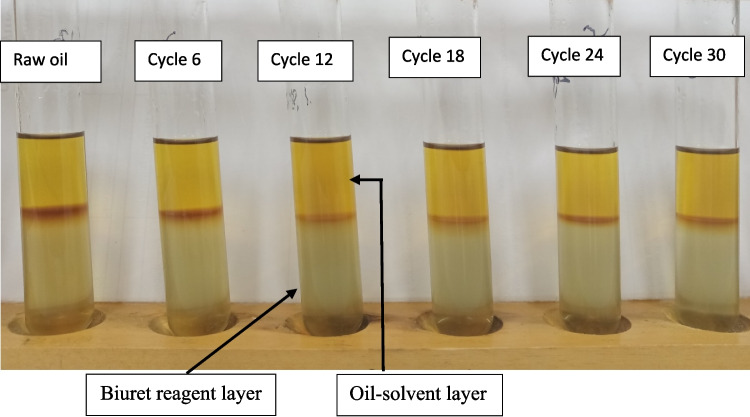
Modified Biuret Protein test to observe the presence of enzyme in the esterified oil product after multiple cycles. Images show reagent colour in oil samples

The modified Biuret test was used to provide a qualitative indication of protein in the esterified squid viscera oil. Figure 7 shows two distinct layers in each test tube, with an upper oil-solvent layer and a lower protein/reagent layer. A blue reagent layer indicates the presence of protein. A constant colour intensity and lack of any blue in each layer through the cycles indicated that the enzyme did not migrate into the oil and was generally retained in the immobilised phase.

### Fatty acid composition of re-esterified squid oil

The fatty acid compositions of the re-esterified squid oil after cycles 12, 18, 24 and 35 were determined and compared to the crude oil. There were no apparent changes observed after any of these cycles compared to the input oil (Table 1). The squid oil has a high content of polyunsaturated fatty acid (PUFA, 45.37%) and lower concentrations of monounsaturated fatty acid (MUFA, 26.21%) and saturated fatty acid (SFA, 24.88%). Levels of EPA (19.98%) and DHA (19.92%) were consistent up to cycle 35.

**Table 1:** Fatty acid compositions of the squid oil (before and after esterification). For all fatty acids there were no significant differences (all p-values > 0.05) between the input oil and products after each cycle analysed

Fatty acid components	% Fatty acids (results are mean values of three replicates)				
	Input oil	C 12	C 18	C 24	C 35
Myristic acid, C14:0	4.75 ± 0.7	4.61 ± 0.6	4.66 ± 0.7	4.46 ± 0.7	4.79 ± 0.5
Tetradecanoic acid, C14:1n5c	0.52 ± 0.07	0.51 ± 0.03	0.53 ± 0.04	0.50 ± 0.07	0.51 ± 0.05
Pentadecylic acid, C15:0	0.49 ± 0.08	0.44 ± 0.07	0.46 ± 0.03	0.44 ± 0.04	0.47 ± 0.07
Palmitic acid, C16:0	13.38 ± 1.8	12.19 ± 1.6	12.61 ± 1.5	10.94 ± 1.6	13.01 ± 1.8
Palmitoleic acid, C16:1n7c	5.38 ± 0.5	5.52 ± 0.7	5.48 ± 0.4	5.57 ± 0.7	5.50 ± 0.4
Stearic acid, C18:0	1.71 ± 0.2	1.54 ± 0.3	1.54 ± 0.2	1.38 ± 0.1	1.72 ± 0.08
Oleic acid, C18:1n9c	16.33 ± 1.6	16.82 ± 1.5	16.78 ± 1.4	17.00 ± 1.8	16.53 ± 1.5
Linoleic acid (LA), C18:2n6c	2.42 ± 0.5	2.53 ± 0.3	2.50 ± 0.4	2.54 ± 0.5	2.35 ± 0.2
α-Linoleic acid (ALA), C18:3n3	1.75 ± 0.4	1.79 ± 0.5	1.76 ± 0.3	1.82 ± 0.2	1.71 ± 0.5
Arachidic acid, C20:0	4.55 ± 0.4	4.61 ± 0.7	4.65 ± 0.5	4.74 ± 0.6	4.57 ± 0.7
Eicosenic acid, C20:1n9	3.98 ± 0.5	4.18 ± 0.7	4.25 ± 0.5	4.28 ± 0.7	4.33 ± 0.3
Arachidonic acid, C20:4n6	1.29 ± 0.2	1.84 ± 0.2	1.35 ± 0.3	1.78 ± 0.5	1.38 ± 0.2
Eicosapentaenoic acid, (EPA) C20:5n3	19.98 ± 1.8	20.61 ± 1.7	20.76 ± 1.6	21.19 ± 1.5	20.37 ± 1.6
Docosahexaenoic acid, (DHA) C22:6n3	19.92 ± 1.6	20.66 ± 1.7	20.87 ± 1.8	21.35 ± 1.5	20.68 ± 1.8
**Saturated fatty acids (SFA)**	**24.88** ± 1.9	**23.40** ± 2.1	**23.93** ± 1.8	**21.97** ± 1.7	**24.57** ± 2.0
**MUFA**	**26.21** ± 1.7	**27.02** ± 1.8	**27.04** ± 1.9	**27.35** ± 1.9	**26.85** ± 2.1
**PUFA**	**45.37** ± 2.8	**47.44** ± 2.1	**47.23** ± 2.9	**48.67** ± 2.5	**46.50** ± 3.1

## Discussion

Squid oil viscera is known to be naturally high in FFA, most likely due to the action of endogenous enzymes in the viscera. Lipid class levels in our crude oil were consistent with those previously reported (Kim et al. 1997), where squid viscera oil from *Illex argentinus* was found to contain approximately 43.0% TAG, 28.0% FFA, 7.5% DAG and 5.5% MAG. The principal objective of the enzymatic esterification in this study is used to convert the high FFA viscera oil into a product containing greater than 95% acylglycerols through reaction of FFA with glycerol, including glycerol of the DAG and MAG (Haque et al. 2024). The demonstrated reusability of the immobilised lipase RMIM for 35 cycles of re-esterification in this study represents a significant improvement in viability on previous work showing typical stability over 10 to 20 reaction cycles when treating other fish and omega-3 oils (Liu et al. 2024; Marín-Suárez et al. 2019). For example, reusing the immobilised lipase for 35 cycles reduces the input enzyme costs to just 2.9% of the calculated costs for replacing the enzyme at every cycle. Assuming a Lipozyme RMIM cost of $US 650/kg and 1% m/v loading, over 35 runs the cost of enzyme equates to US$0.19 per litre of squid oil product, compared to US$0.65 per litre if the enzyme is replaced every 10 cycles.

The greater stability is most likely due to our custom designed reactor which separates the enzyme from impact with the impellor blades for stirring (Haque et al. 2024; A. Joshi et al. 2025). Additionally, the lack of a washing step between each cycle prevents formation of a hydrophobic or hydrophilic hinderance around the enzyme particles (Keng et al. 2008). The reaction media were completely drained from the enzyme between cycles to ensure activity was not adversely affected by a lack of washing.

The enzyme is driven through several steps to reach an irreversible denatured state by external stresses. A two-step denaturation is well defined in the literature (Parris and Baginski 1991; Sava et al. 2005). In the first step, the protein partially unfolds at a relatively low-stress level and loses its tertiary structure. This was observed by a decline in performance as determined by free fatty acid yield during the initial esterification (up to cycle 14). From cycles 15 to 23, a significant amount of enzyme was partially unfolded and flexibly attached to the fractured or partially broken carrier, forming hydrophobic pockets binding with the ANS to yield increased fluorescence emission. This confirms the molten globular state where the enzymes lack tight packing, flexibility and readiness for reaction (Haque et al. 2013; Parris and Baginski 1991). The more reactive flexible state of the enzyme likely led to an enhanced conversion performance within this cycle range.

In the second step, a complete unfolding of protein takes place if the stress is continued. The reduced fluorescent emission at 35 cycles suggests irreversible denaturation of the enzyme. The completely unfolded polypeptides ultimately form protein aggregates. An increase in unfolded groups promotes the formation of communal aggregates, which reduces the number of free hydrophobic groups, producing lower hydrophobic emission (Park et al. 2011; Ptitsyna and Uversky 1994). This aggregated state is characteristic of irreversible denaturation when these biomolecules lose normal functionality. Structural changes were also noted after an increase in the number of cycles.

Morphological changes observed by particle breakage and changes in enzymatic activity have been previously reported by (Bolivar et al. 2022; Sabbani et al. 2006). We observed the tight immobilised resin core (in the original particle) gradually loosening and breaking as the cycle number increased. This particle breakage reduces the particle size which can facilitate improved mass transfer, possibly leading to the apparent increase in bioactivity between successive reuses observed in this study from cycles 14 to 23 and described in (Bolivar et al. 2022). By cycle 35, the proportion of enzyme spheres with thinner coating had increased, with numerous broken resin particles and fragmented cores. This irreversible degradation accounted for the significant loss in acylglyceride yield observed. Black clot-like formations in the images were potentially a result of coagulation of the detached enzymes from the resins.

The general change in secondary structure showed an α-helix and β-sheet transition over cycles which is consistent with unfolding and followed by aggregation. No suitable direct studies were found to compare this with other reports of Lipozyme RMIM in the literature; however, this composition is consistent with values for immobilised lipase B from *Candida antarctica*, CALB, (∼31% α-helix and ∼19% β-sheet) and the recombinant *Candida rugosa* lipase (23.80% α-helix and 23.40% β-sheet) (Mei et al. 2003; Natalello et al. 2005). Previous work by (Tatham et al. 1985) reports the β-turns are flexible structures that work in folding the polypeptides and play an essential role in the orientation of α-helix and β-sheet. Due to the flexible and unstable nature of β-turns and random coil, these two structural elements changed quicker than α-helix and β-sheet which are closely bonded by several amino acids present at their backbones. Therefore, in the current study, the β-turns content decreased as reaction cycles progressed, whereas the α-helix and β-sheet contents increased. The fatty acid compositions of the squid oil product are comparable to values previously reported (Asadpour 2016), where PUFA/MUFA/SFA contents were found to be 40.2 ± 2%, 23.7 ± 3% and 29.4 ± 2%, respectively. The esterification of squid oil over increasing number of enzyme cycles did not change this ratio of PUFA/MUFA/SFA significantly (*p* > 0.05), indicating that the omega-3 fatty acids are stable. Levels of EPA and DHA are consistent with levels previously observed in viscera oil (*p* > 0.05) (Kang et al. 2005) over each of the 35 cycles studied.

Overall, our findings show progressive loss of activity was linked to both enzyme denaturation and carrier breakdown. Resin breakdown was a dominant factor after a higher number of cycles, indicating that modifying reactor design to further restrict carrier movement could provide useful gains in enzyme reusability. In this study, the removal of enzyme for sampling likely increased movement within the enzyme compartment. This suggests the lifespan of immobilised RMIM may be underestimated in this work. Furthermore, a more mechanically robust food grade carrier could enable further reuse cycles.

## Conclusions

Immobilised Lipozyme RMIM is suitable for at least 30 repeat cycles for conversion of free fatty acids to triacylglycerides. Yield remained at over 90% of starting conversion throughout the 30 cycles and decreased to 86.14% after 35 cycles. Reusing the enzyme for 35 cycles reduces the cost to just 2.9% compared to replacing the catalyst every cycle, a significant improvement for large-scale viability. Changes in lipase performance were attributed to both enzyme denaturation and carrier breakdown. Enzyme denaturing appears to progress through a transition from α-helix and β-sheet structures, with subsequent aggregation. No loss of enzyme into the oil was observed and although there were significant enzyme structure and resin changes, activity was mostly retained over the first 32 cycles. The outcomes of this study can guide improvements to reactor design to reduce mechanical stress on the enzyme upon process development and scale-up. 

## Data Availability

Data will be provided upon request.
